# Broadening the application of Yarrowia lipolytica synthetic biology tools to explore the potential of Yarrowia clade diversity

**DOI:** 10.1099/mic.0.001472

**Published:** 2024-06-24

**Authors:** Young-Kyoung Park, Tristan Rossignol

**Affiliations:** 1 Université Paris-Saclay, INRAE, AgroParisTech, Micalis Institute, 78350, Jouy-en-Josas, France

**Keywords:** carotenoids, Golden Gate, synthetic biology, Yarrowia clade, Yarrowia lipolytica

## Abstract

Yeasts have established themselves as prominent microbial cell factories, and the availability of synthetic biology tools has led to breakthroughs in the rapid development of industrial chassis strains. The selection of a suitable microbial host is critical in metabolic engineering applications, but it has been largely limited to a few well-defined strains. However, there is growing consideration for evaluating strain diversity, as a wide range of specific traits and phenotypes have been reported even within a specific yeast genus or species. Moreover, with the advent of synthetic biology tools, non-type strains can now be easily and swiftly reshaped. The yeast *Yarrowia lipolytica* has been extensively studied for various applications such as fuels, chemicals, and food. Additionally, other members of the *Yarrowia* clade are currently being evaluated for their industrial potential. In this study, we demonstrate the versatility of synthetic biology tools originally developed for *Y. lipolytica* by repurposing them for engineering other yeasts belonging to the *Yarrowia* clade. Leveraging the Golden Gate *Y. lipolytica* tool kit, we successfully expressed fluorescent proteins as well as the carotenoid pathway in at least five members of the clade, serving as proof of concept. This research lays the foundation for conducting more comprehensive investigations into the uncharacterized strains within the *Yarrowia* clade and exploring their potential applications in biotechnology.

## Introduction

Bio-based production through the microbial fermentation process to replace petroleum-based chemical production is extensively studied as an essential way to develop sustainable and environmentally friendly bioproduction. A key point for microbial cell factory is the choice of the chassis microorganism for its metabolite production and its metabolic rewiring capacity as well as the availability of synthetic biology tools. Yeasts are particularly attractive eukaryotic production hosts due to their capacity to grow on a broad variety of renewable carbon sources, to produce complex target molecules, and their well-established scale-up process.

Apart from *Saccharomyces cerevisiae*, non-conventional yeasts have been investigated due to their particular industrial traits. Among them, *Yarrowia lipolytica* has been extensively studied as a chassis for many applications from fuels and chemicals to foods. This yeast is genetically amenable, can grow on a large range of substrates, has the GRAS (Generally Regarded as Safe) status, and can produce high yields of various biomolecules, in particular lipids and organic acids [1]. Target molecules produced by this yeast have been further expanded to plant-based derivative products like terpenes [2]. This continuously growing interest in using this yeast as an industrial chassis has led to an exponential advancements in the development of synthetic biology tools including fast and modular cloning systems and CRISPR/Cas9 methods [3].

Yi *et al.* recently reviewed the importance of considering strain diversity for establishing microbial cell factories by highlighting the fact that a wide range of strain variations already exists even within a specific yeast genus and species [4]. An illustrative example has been provided by a primary screening of the potential producer of triacetic acid lactone by expressing a 2-pyrone synthase in 13 industrial *S. cerevisiae* strains with different genetic backgrounds [5]. The production levels varied up to 63-fold among different strains, highlighting the importance of considering the strain variation for optimal microbial cell factories. Moreover, basal production does not consistently signal the potential for rewiring, and there are variations in phenotypes and physiology when it comes to non-native metabolites [4]. Therefore, heterologous expression must be screened in various backgrounds to evaluate the best strains/species for further genetic engineering. This emphasizes the growing importance of considering strain-level characteristics when selecting host microorganism. However, this requires extensive study of variants or screening with the requirements of genetic tools adaptations to each strain.

Most studies focus on a limited set of industrial or type strains and rarely extend to other strains or related species with limited knowledge on the physiological and metabolic state of such species or strains. Nevertheless, for some interesting traits like lipid production, physiological data of clade or set of strains have been explored. The diversity of closely related species in the *Yarrowia* clade has been investigated through comparative studies of their oleaginous properties [6]. These species including *Y. lipolytica*, *Yarrowia alimentaria*, *Yarrowia deformans*, *Yarrowia galli*, *Candida hispaniensis*, *Yarrowia hollandica*, *Yarrowia oslonensis*, *Yarrowia phangngensis* and *Yarrowia yakushimensis* which have exhibited different levels of tolerance to salt, different optimal temperatures for growth [7], maximum lipid content, and cell dry weights ranging from 30 % in *C. oslonensis* to 67 % in *C. hispaniensis* [8]. In the past decade, the clade has grown up to 15 potential species including *Yarrowia keelungensis*, *Yarrowia divulgata*, *Yarrowia porcina*, *Yarrowia bubula*, *Yarrowia brassicae* and *Yarrowia parophonii* [9,11]. Quarterman *et al.* investigated the biomass and lipid production of 13 species of the *Yarrowia* clade by using a non-detoxified acid-pretreated switchgrass hydrolysate as a feedstock [9] with further characterization of a subset of them for inhibitor tolerance. This study highlighted the diversity in terms of behaviour, production and resistance, for example, *Y. hollandica* and *Y. phangngensis* reached higher cell biomass and lipid titre compared to *Y. lipolytica* W29 in these conditions. In other studies, up to thirteen of these yeast species from the *Yarrowia* clade were screened for erythritol, arabitol, mannitol, citric acid, lipids, and protein production [12,13]. In particular, *Y. divulgata* and *Y. oslonensis* were identified as robust producers of polyol compared to *Y. lipolytica* when grown on glycerol [13]. Protease production among the *Yarrowia* clade has been confirmed the potential of certain *Yarrowia* species to secrete significantly high amounts of proteases and revealed the influence of culture media when benchmarking species for a particular trait [14].

Therefore, the physiological aspect and diversity of the *Yarrowia* clade are now well-documented, highlighting the variation in industrially relevant traits among different species, which can outperform *Y. lipolytica*. Following this, genetic engineering of the other species in the clade starts to be considered. A first transformation method for the most distant species *C. hispaniensis* allows using sucrose as a carbon source by expressing the *S. cerevisiae* invertase as a marker and a heterologous enzyme [15]. * C. hispaniensis* is resistant to antibiotics like gentamycin, nourseothricin, and hygromycin, which are typically employed as selective markers in yeast genetic engineering. This limits the development of genetic tools in this species [15]. Moreover, this yeast is refractory to the standard transformation method and requires the use of biolistic approach for transformation [15]. Similarly, * Y. phangngensis* has been transformed for the first time by using genetic tools and the standard LiAc/PEG transformation method neutralizing the cell membrane by lithium cations developed for *Y. lipolytica* with hygromycin and zeocin as markers for selections [16]. The recycling of *Y. lipolytica* tools in this species shows possibility for more complex genetic engineering. However, it is worth noting that the hygromycin concentration required for selection in *Y. phangngensis* is three to five times higher compared to *Y. lipolytica* transformants selection. It opens the way for reusing the existed tools or developing the standardized tools in non-conventional and non-type organisms, allowing rapid screening of numerous potential hosts for diverse applications.

Here we evaluated the functionality of a Golden Gate modular synthetic biology tool developed for *Y. lipolytica* [17] in six species from the *Yarrowia* clade by using two different selection markers and the expression of a fluorescent protein as a validation system with a fast transformation method. As a proof of concept, the genes in the carotenoid pathway were expressed in five of the six species.

## Methods

### Strains and media

All the wild-type yeast strains used in this study are listed in Table 1. All constructed strains are listed in Table S1, available in the online version of this article.

Chemically competent *Escherichia coli* DH5α cells were used for cloning and plasmid propagation. *E. coli* cells were grown at 37 °C with constant shaking on 5 ml of LB medium with ampicillin (100 µg ml^−1^) or kanamycin (50 µg ml^−1^) for plasmid selection. Yeasts were grown on rich medium YPD (10 g l^−1^ of yeast extract, 10 g l^−1^ of peptone, 10 g l^−1^ of glucose) complemented with hygromycin (200 µg ml^−1^) or nourseothricin (500 µg ml^−1^) for the selection of transformants. Solid media were prepared by adding 1.5 % agar. For growth on 96-well plates, yeasts were grown on YNB minimal medium containing 10 g l^−1^ glucose, 1.7 g l^−1^ yeast nitrogen base without amino acids, 5.0 g l^−1^ NH_4_Cl and 50 mM phosphate buffer (pH 6.8).

### Plasmid construction

Integrative plasmids expressing the Redstar2 fluorescent protein were assembled by using Golden Gate method as described in our previous study [18] containing hygromycin or nourseothricin marker, pTEF promoter, tLip1-3 terminator, ZETA upstream and downstream integration sequences, Redstar2 gene and pSB1A3 backbone vector. All building parts are described in Larroude *et al*. [17].

Plasmid GGE115 allowing expression of the carotenoid pathway was described in Larroude *et al.* [18]. It contains the ZETA sequences for random integration, the hygromycin marker, and the expression cassette of three genes involved, GGS1 (geranylgeranyl diphosphate synthase) from *Y. lipolytica*, carPR (phytoene synthase/lycopene cyclase) from *Mucor circinelloides*, and carB (phytoene dehydrogenase) from *M. circinelloides* [19].

### Promoter sequences alignment

The *Y. lipolytica* pTEF promoter was used to retrieve promoter ortholog from Whole Genome Sequences of the other species (*Yarrowia_osloensis* ULGU01000000; *Yarrowia_deformans* ULGY01000000; *Yarrowia_galli* ULGS01000000; *Yarrowia_yakushimensis* ULGW01000000; *Yarrowia_alimentaria* ULGN01000000; *Yarrowia_hollandica* ULGV000000). Multiple sequences alignment was performed using T-Coffee web server [20]

### Transformation

EZ-Yeast transformation kit (MP Biomedicals) was used for yeast transformations following the manufacturer’s instruction. Transformation mix with the cell was incubated for 30 min at 39 °C. Plasmids were first digested with restriction enzyme NotI to release the expression cassette and 2 µg of digested DNA was used for transformation. For the release of the expression cassette of plasmid GGE115 the restriction enzyme SfiI was used.

### Growth curves and fluorescence measurements

Yeasts were precultured for 24 h in YNB medium and diluted to an OD_600nm_ of 0.1 in fresh YNB medium and 200 µl was transferred into 96-well microplates. The growth analysis was performed using a microtitre plate reader (Synergy Mx; BioTek). The settings were 28 °C and constant shaking with a reading OD_600nm_ and red fluorescence (excitation 558 nm /emission 586 nm) every 30 min for 72 h. In the results, fluorescence was calculated as a ratio of relative fluorescence to OD_600nm_. Cultures were performed at least in duplicate.

### Carotenoids measurements

Yeasts were precultured for 24 h in YPD medium and diluted to an OD_600nm_ of 0.1 in fresh YPD medium with 5 % glucose concentration. Cells were grown in flask for 5 days in duplicates. Cell pellets from 100 µl culture broth were collected in 2 ml screwcap microtubes and washed with distilled water. Then 200 µl acid-washed glass beads and 400 µl acetone were added to the pellets and carotenoids were extracted by lysing cells with a FastPrep (MP biomedical) set with three cycles at 4500 r.p.m. for 30 s. The extract was collected by centrifugation at 12000 r.p.m. for 3 min, and the procedure was repeated until the pellet and the supernatant were colourless. Carotenoids concentration was evaluated using a SAFAS spectrophotometer at 448 nm using β-carotene standard (Sigma 22040) for calibration curve. T-test were performed using biostatTGV between YL_006 and the other strains. *P*-values lower than 0.05 were considered significant.

## Results

### Evaluation of selection markers

The Golden Gate tool kit developed for *Y. lipolytica* containing two dominant selection markers, hygromycin and nourseothricin, was used in this study. This toolkit enables the expression of up to three transcription units in a single transformation [17]. The minimum concentration of hygromycin and nourseothricin required for growth inhibition was first evaluated on eight strains of the *Yarrowia* clade (*Y. lipolytica*, *Y. alimentaria*, *Y. deformans*, *Y. galli*, *Y. hollandica*, *Y. oslonensis*, * Y. phangngensis* and *Y. yakushimensis*) [8]. All the strains used in this study are listed in Table 1. *C. hispaniensis* was excluded due to its resistance to the major antibiotics [15] and the available antibiotic markers in the *Y. lipolytica* Golden Gate toolkit would not be effective for this species. The growth of all the strains were inhibited with 200 µg ml^−1^ hygromycin or 500 µg ml^−1^ nourseothricin except for *Y. phangngensis* which required higher concentration as already demonstrated [16]. In our hands, we had residual growth of *Y. phangngensis* with 500 µg ml^−1^ of hygromycin and 1 mg ml^−1^ of nourseothricin. Therefore, this species was not selected thereafter for evaluation of transformability with the *Y. lipolytica* Golden Gate plasmids.

**Table 1.** List of wild-type yeast strains of the *Yarrowia* clade used in this study. IDs correspond to internal INRAE yeast collection number. CBS: Fungal and yeast collections in Westerdijk Fungal Biodioversity Institute. W29 from own collection

ID	Yeast strain
Y2183	*Yarrowia lipolytica* W29
Y8112	*Yarrowia oslonensis* CBS 10146
Y8113	*Yarrowia deformans* CBS 2071
Y8114	*Yarrowia galli* CBS 9722
Y8115	*Yarrowia phangngensis* CBS 10407
Y8116	*Yarrowia yakushimensis* CBS 10253
Y8117	*Yarrowia alimentaria* CBS 10151
Y8119	*Yarrowia hollandica* CBS 4855

### Evaluation of transformation capacity

To evaluate the transformation of each strain, we used integrative expression vectors assembled by the Golden Gate method. These harbour the expression cassette of the Redstar2 fluorescent protein, the selective marker either hygromycin or nourseothricin, and ZETA sequences (retrotransposon of *Y. lipolytica*, *YLT1*) that are known for random integration into the * Y. lipolytica* genome (see Methods). All the building blocks have been optimized for *Y. lipolytica* and the details are described in Larroude *et al.* [17]. The two genes coding Redstar2 and antibiotic resistance were expressed under the *Y. lipolytica* pTEF promoter. Therefore, we first evaluated the level of homology between the pTEF promoter sequences among the different strains of the clade. The *Y. lipolytica* pTEF promoter was used to retrieve promoter ortholog from Whole Genome Sequences of the other species. All sequences retrieved are available in Supplementary File 1. Sequence alignment showed a strong homology, particularly in the 220 base pair region proximal to the start codon, which suggests the potential for cross-species functionality of the promoter. This region corresponds mainly to the pTEF core promoter identified *in Y. lipolytica* while the region with low homology corresponds to putative upstream activation sequences [21]. This low homology may reveal some putative differences in the regulation of the pTEF promoter between species. *Y. alimantaria* appears the most distant to *Y. lipolytica* and is missing a homology region in the distal part of the promoter (Supplementary File 2).

The transformation of seven species including *Y. lipolytica* as a positive control was carried out by using a simple and rapid method with a yeast transformation kit (see Methods section), in line with the objectives of rapid screening of different hosts without specific transformation setups. Both plasmids expressing the Redstar2 with hygromycin or nourseothricin were tested. We were able to obtain from one to around 50 clones for all strains in one transformation experiment except for *Y. alimentaria*. Despite several attempts, we could not obtain any transformants of *Y. alimentaria* even with an extended incubation time exceeding 1 week. The relatively lower promoter sequence homology between *C. alimentaria* and *Y. lipolytica,* compared to other species within the clade, might explain the poor expression of selective marker genes resulting in unsuccessful transformation. Individual clones from all transformants were then evaluated for their fluorescence in a microtitre plate reader. All the strains constructed are listed in Table S1, available in the online version of this article.

**Fig. 1.**
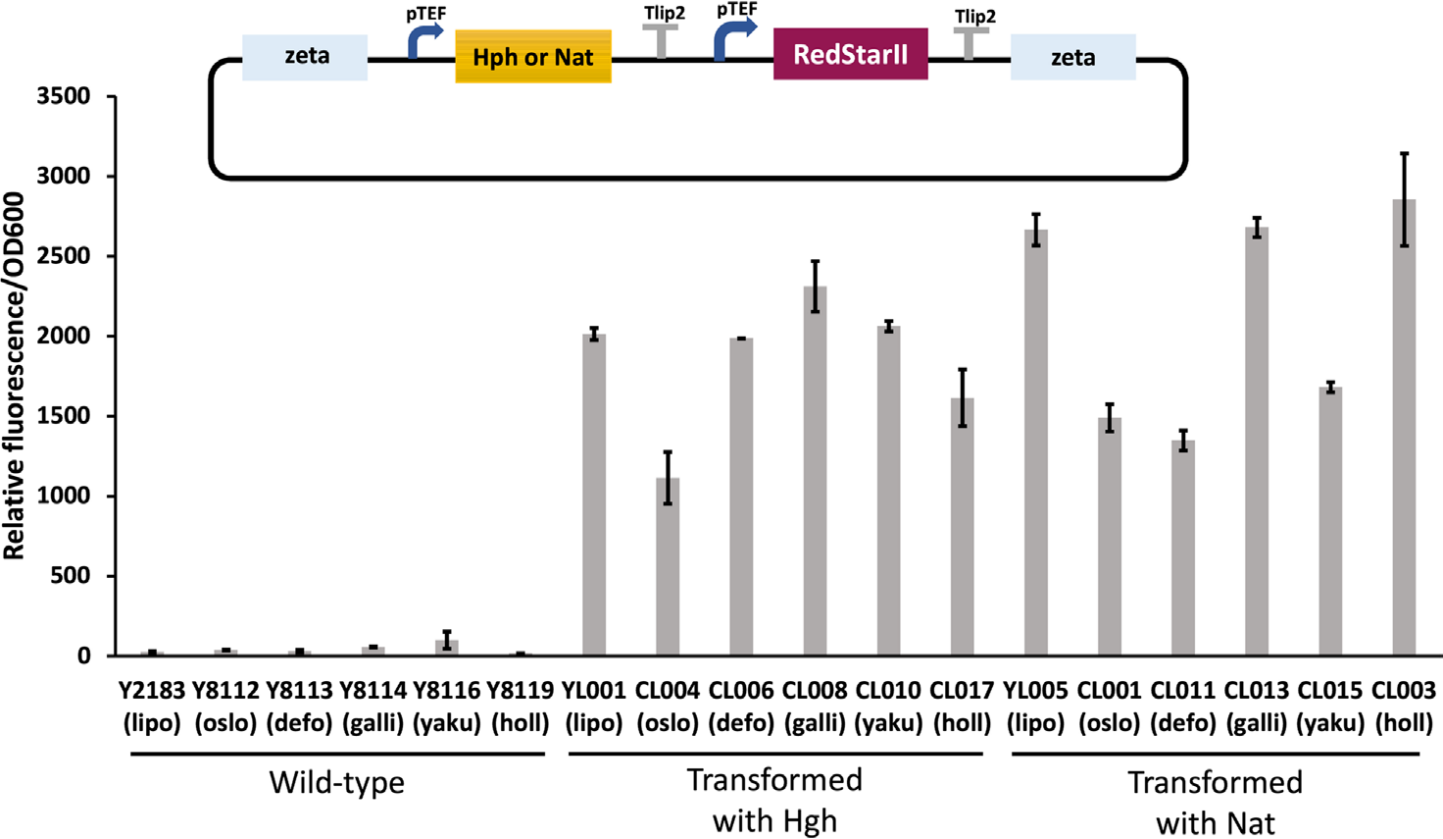
Relative fluorescence to OD for wild-type strains and each transformant expressing the Redstar2 protein after 72 h of cultivation in 96-well microplates. Cultures were performed at least in duplicate. The upper illustration corresponds to the vector used for transformation. Hgh (vector Hygromycin –pTEF-Redstar2); Nat (vector Nourseothricin –pTEF-Redstar2). lipo (*Y. lipolytica*); oslo (*Y. oslonensis*); defo (*Y. deformans*); galli (*Y. galli*); yaku (*Y. yakushimensis*); holl (*Y. hollandica*). All the strains are listed in Table S1. Error bars correspond to standard deviations.

**Fig. 2.**
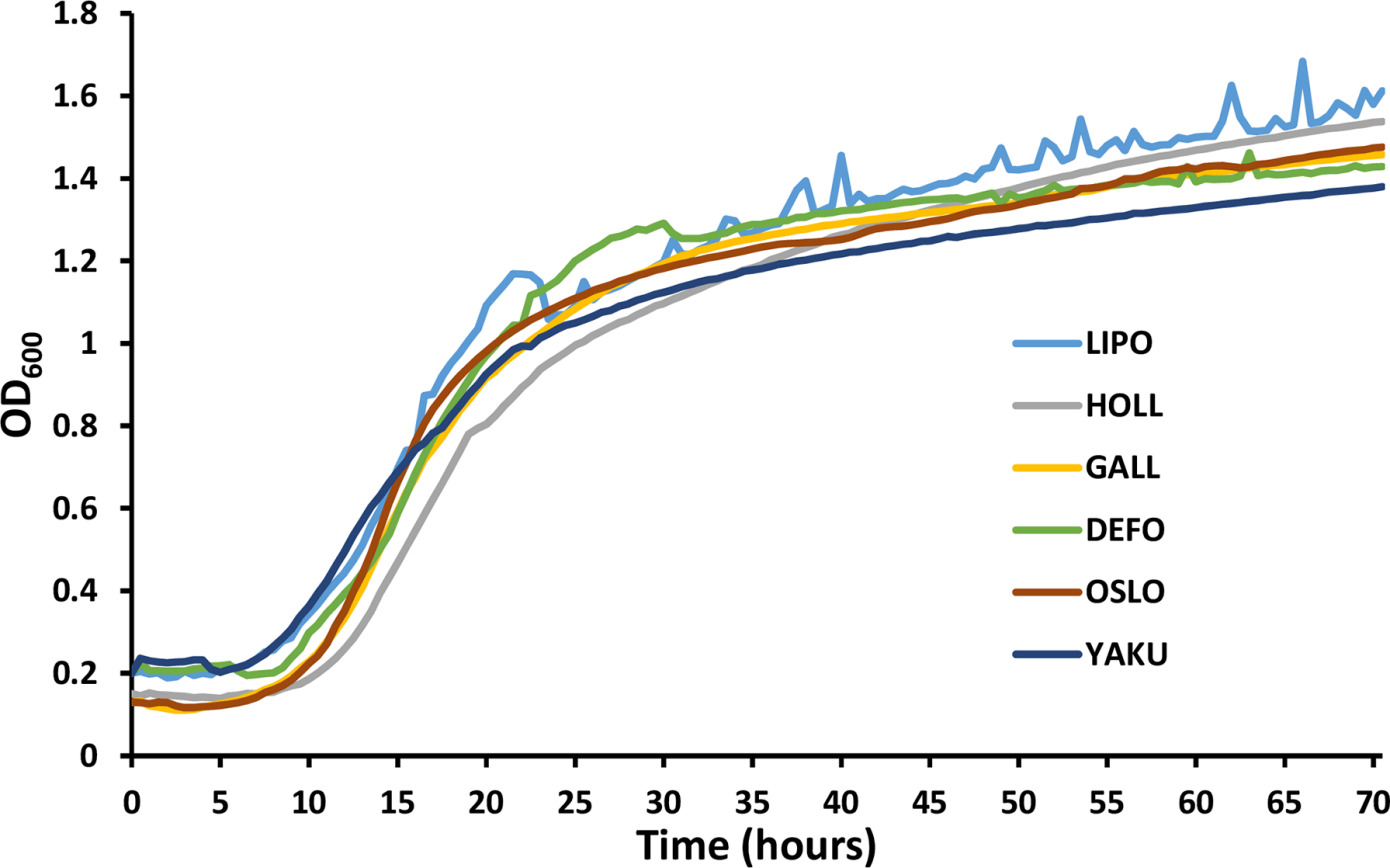
Representative growth curves in 96-well plates for each species. Growth was performed in triplicate. Only one curve for each strain is represented for clarity. Strains were grown in 200 µl of YNBD minimal medium containing 10 g l^−1^ glucose, 1.7 g l^−1^ yeast nitrogen base without amino acids, 5.0 g l^−1^ NH_4_Cl and 50 mM phosphate buffer (pH 6.8) at 28 °C for 72 h. The growth analysis was performed using a microtitre plate reader with a reading every 30 min. LIPO (*Y. lipolytica*); OSLO (*Y. oslonensis*); DEFO (*Y. deformans*); GALL (*Y. galli*); YAKU (*Y. yakushimensis*); HOLL (*Y. hollandica*).

All the clones tested were expressing the Redstar2 fluorescent protein with some variation depending on the species and marker. *Y. lipolytica* and *Y. galli* were the species expressing the highest levels of fluorescence on average with both markers while *Y. oslonensis* was the lowest (Fig. 1). Those differences are not due to the variation in fitness between species as they present a similar growth rate (Fig. 2). Final OD_600_ at 72 h ranges between 1.4 and 1.61 and therefore does not have a major impact on the fluorescence to OD differences observed in Fig. 1. As the genomic integration takes place randomly, we cannot formally compare the level of fluorescence as genome integration locus may influence the level of heterologous expression. In future study, controlling the copy number through site-specific integration will be helpful to compare the expression levels across the *Yarrowia* clade.

### Heterologous pathway expression

**Fig. 3.**
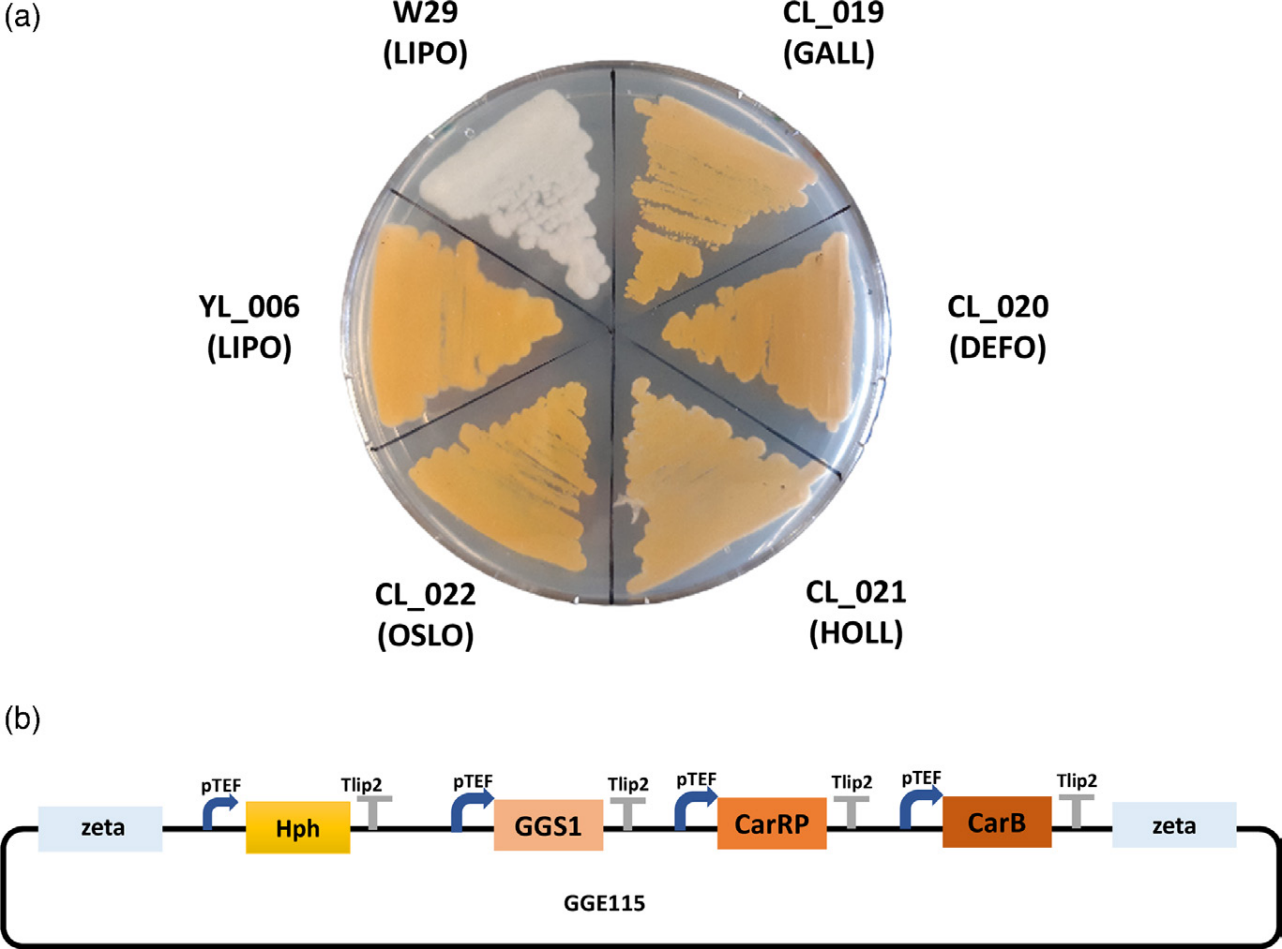
**a**) Growth on YNB plates of strains transformed with the GG115 plasmid for carotenoid pathway expression. LIPO (*Y. lipolytica*); OSLO (*Y. oslonensis*); DEFO (*Y. deformans*); GALL (*Y. galli*); HOLL (*Y. hollandica*); W29 (*Y. lipolytica* wild-type strain without transformation). All the strains are listed in Table S1. **b**) Schematic draw of the vector GGE115 used for transformation.

**Fig. 4.**
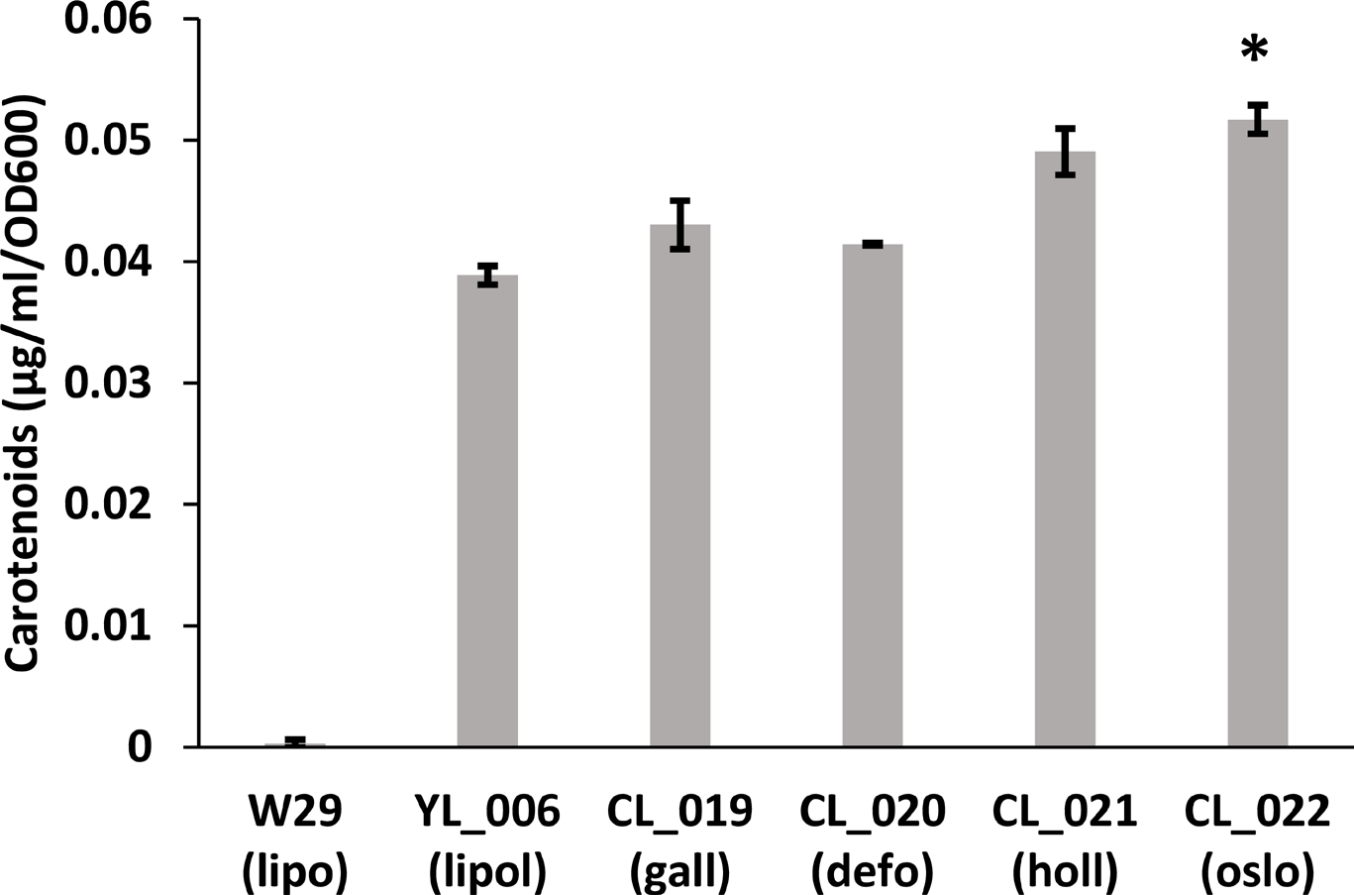
Carotenoids production in micrograms per millilitre of culture normalized per OD after 5 days of growth. lipo (*Y. lipolytica*); oslo (*Y. oslonensis*); defo (*Y. deformans*); gall (*Y. galli*); holl (*Y. hollandica*); W29 (*Y. lipolytica* wild-type strain without transformation). All the strains are listed in Table S1. Error bars correspond to standard deviations of two biological replicates. * Statistically significant difference between a clade species and YL_006 (t-test *p*-value<0.05).

The evaluation was pushed forward to express a heterologous pathway previously established in *Y. lipolytica* for carotenoid production [18] by using a plasmid containing three transcription units (see Methods section). This pathway is often used as a proof of concept as it allows rapid screening of positive clones with the orange colour. This heterologous pathway-expressing cassette was transformed into seven species, respectively. As we have seen from the transformation of Redstar2 gene expression cassette, we were not able to obtain transformants for *Y. alimentaria*. Additionally, we were not able to obtain transformants for *Y. yakushimensis*. Except for these two species, we observed transformants with a deep orange colour for the species tested in this study (Fig. 3). All the transformants are listed in Table S1. Again, we observed slight differences in colour intensity among species. In order to quantify differences in carotenoids production, we grew transformants in rich medium for 5 days, extracted carotenoids, and measured their concentrations (Fig. 4). We observed differences between species, with a significant 32 % increase between *Y. lipolytica* (YL_006) and *Y. oslonensis* (CL_022) with a higher production for the latter while their growths were similar. These production differences are the opposite trend of what we observed for Redstar2 production (Fig. 1). The differences in the levels of carotenoid synthesis could either be due to differences in heterologous expression or metabolic capacity among species for this particular pathway, or the integration locus affecting native metabolism that may connect with carotenoid synthesis. The fact that we have different trends between fluorescent protein expression and carotenoid pathway expression from the same strain is in favour of a metabolic capacity variability.

## Conclusion

In this study, we demonstrate that the synthetic biology tools developed for the yeast *Y. lipolytica* can be effectively repurposed for genetic engineering of other members of the clades, in particular for *Y. oslonensis*, *Y. deformans*, *Y. galli*, and * Y. hollandica* for which we were able to express a multi-gene heterologous pathway. *Y. phangngensis* and *C. hispaniensis* exhibit a distinct GC content compared to other members of the clades and significant reduction in size for some protein families [7], indicating a greater sequence divergence. Furthermore, both strains are highly resistant to typical antibiotics, which may suggest lower compatibility, if any, with *Y. lipolytica*’s dedicated tools. On the other hand, *Y. alimentaria* is much closer *to Y. lipolytica* in terms of GC content, and its protein expression failure could help to determine the homology threshold for compatible expression, especially for promoters, although further investigation is required.

While it is widely recognized that the choice of host is crucial in metabolic engineering applications, there is still limited understanding of the strain diversity among non-type strains, particularly for non-conventional microorganisms. Therefore, exploring this aspect of diversity is a critical route toward achieving success in microbial cell factories. By evaluating other relevant traits, we anticipate that the results presented in this study will facilitate new biotechnological applications involving strains within the *Yarrowia* clade that were previously neglected and not thoroughly characterized.

## supplementary material

10.1099/mic.0.001472 Uncited Fig. S1.
