# Supplementary material for: Broadening the application of Yarrowia lipolytica synthetic biology tools to explore the potential of Yarrowia clade diversity
Source: Microbiology (Reading). 2024 Jun 24;170(6):001472. doi: 10.1099/mic.0.001472 (PMC11261841; doi:10.1099/mic.0.001472)
Supplement: Uncited Fig. S1. [file mic-170-01472-s001.pdf]

Supplementary file 1

>pTEF_Yarrowia_lipolytica_goldengate system
GGGTTGGCGGCGTATTTGTGTCCCAAAAAACAGCCCCAATTGCCCCAATTGACCCCAAATTGACCCAGTAGCG
GGCCCAACCCCGGCGAGAGCCCCCTTCACCCCACATATCAAACCTCCCCCGGTTCCCACACTTGCCGTTAAGG
GCGTAGGGTACTGCAGTCTGGAATCTACGCTTGTTCAGACTTTGTACTAGTTTCTTTGTCTGGCCATCCGGGT
AACCCATGCCGGACGCAAAATAGACTACTGAAAATTTTTTTGCTTTGTGGTTGGGACTTTAGCCAAGGGTATA
AAAGACCACCGTCCCCGAATTACCTTTCCTCTTCTTTTCTCTCTCTCCTTGTCAACTCACACCCGAA

>Yarrowia_osloensis ULGU01000001.1:3128419-3128825 Yarrowia osloensis strain CBS 10146 genome assembly, contig: YAOS0S01, whole genome shotgun sequence reverse complement
GGGTCGTGGAG
GCGATATTTTCGCCCGGCGGAAACACACAATTGCTCCAATTGGCGCCCAATTGACCCTGT
AAACTCCCCAGAGCCCCACACCCGCTCCCTCACCCCACACAACTCGTCTCCCCCGGTTCT
CACACTTGCCGTTAAGGGCGTAGGGTACTGCAGTCTGGAATCTACGCTTGTTCAGACTTC
GGGCCGGTTTCTTTGTCCAGCCATCCGGGTAACCCATGCCGGACGCAAAATAGACTACTG
AAAATTTTTTTGCTTTGTGGTTGGGACTTTAGCCAAGGGTATAAAAGACCACCGTCCCCG
AATTACCTTTCCTCTTTTCTCTCTCTCCTTGTCAACTCACACCTGAA

>Yarrowia_deformans ULGY01000001.1:2134548-2134917 Yarrowia deformans strain CBS 2071 genome assembly, contig: YADE0S01, whole genome shotgun sequence reverse complement
GGTTAGAGTCGCTTAATCGACAATCAAAAACCATACGATTGATCCAA
TTGACGTGCAATTGACCGGGTGGCATTTTCAGAGTCTCACCTTACCTCCCACCCCCACAA
AACCCCGTTTCCCCCACCGTCACACTTGCCGTTAAGGGCGTAGGCAGCTGCAGTCTGGAA
TCTACGCTTGTTCAGAATTCGGACCTTTTTCTTTGTCCGGCCATCCGGGTAACCCATGCC
GACCGCAAAATAGACTACTGAAAATTTTTATGCTCTGTGGTTGGGAGGTAGCCCCAGGGT
ATAAAAGACCACCGTCCCCGAATTACCTTTCCTCTTCTTTTCTCTCTCTCCTTGTCAACT
CACACCCGAA

>Yarrowia_galli ULGS01000006.1:256900-257297 Yarrowia galli strain CBS 9722 genome assembly, contig: YAGA0F, whole genome shotgun sequence
GGGGTTAGAGACTCAATATTGACAATCAAAGT
GGCCAGATTCGTTGCAATTGACCTGCAATTGACCCTGTAGCTTCTCCAGCTCGCCACCACCCCTACATCA
CCCCACACAACACTGTTCCCCCAGTTCTCACACTTGCCGTTAAGGGCGTAGGCGTCTGCAGTCTGGAATC
TACGCTTGTTCAGAATTCAGCCCTGTTTCTTTGTCCAGCCATCCGGGTAACCCATGCCGGACGCAAAATA
GACTACTGAAAATTTTTATGCTCTGTGGTTGGGACTTTAGCCAAGGGTATAAAAGACCACCGTCCCCGAA
TTACCTTTCCTCTTCTTTTCTCTCTCTCCTTGTCAACTCACACCTGAA

>Yarrowia_yakushimensis ULGW01000001.1:4716100-4716600 Yarrowia yakushimensis strain CBS 10253 genome assembly, contig: YAYA0S1, whole genome shotgun sequence reverse complement. ATG find manually en extend sequence
GAGAGTGTTTGAGAGAGAATATTGAGAATGTAAACTCGAGC
AATTGGTCATATTGCGCCCACAATTGACCCTACACGTCTCTCGCAGCCCCAGAACCACCC
AATGACCCCACACAACTCTCTTGCCCCAGTTGTCACACTTGCCGTTAAGGGCGTAGAGTA
CTGCAGTCTGGAATCTACGCATGTTCAGACTTTGGCACTGTTTCTTTGTCTGACGATCCG
GGTAACCCATGCTGGGCGGCAAATAGCCTTTTGAAAATTTTTTTGCCTGGAGGTTGGGAC
TTTGGCCCAGGGTATAAAAGACCACCGTCCCCGAATTACCTTTCCTCTTCTTTTCTCTCT
CTCCTTGTATTAGTTTCATTCAA

>Yarrowia_alimentaria ULGN01000006.1:1167454-1167980 Yarrowia alimentaria strain CBS 10151 genome assembly, contig: YAAL0S06, whole genome shotgun sequence extend sequence
GGCCAAAAGTCGTGC
AGCCGGCAATTCCGCCAATTGACGGCAATTGCGTGCAATTGGACGGCATTTTGCACGCCCAGCCGCCTCC
CCACGTTGCCTACAACCCCACGAGTCCCGCCACCTCTGGGGTCGCTATTGGGGCTTGAGTTTCCCGTTAG

GGCGTAGGGTAATGCAGTCGGTAAGCTACATCCATACAGAGTTTAGTCTCCTTTCTTTGTCCGCTAGGTC
GGGTAACCCATGCTTGTCGCTTTTAGAGTCATGAAAATTTTTTTTTTGCAACACCTGCGTCTTCGCTCCC
AGGTATAAAAGAGACGAGTTTCCGAATTACCTTTCTTCTTTTTTTCTTCTCCTTGTCAACTCACACCTGA
A

>Yarrowia_hollandica ULGV01000013.1:10300-10659 Yarrowia hollandica strain CBS 4855 genome assembly, contig: YAHO0S13, whole genome shotgun sequence
AGACGGCATGTGGCCGAAAGCAGGGCGAAAAACAGTGAATTCCATCCTAATTGAGCACCAATTGACTCTG
TGAGATCCTGAACCACTCATATCACACCCACACCCCACACTCAGCACTCTCCCCGGCACTTCAGTTTCCC
GTTAAGGGCGTAGGGTACTGCAGTCTGGAACCTACGATCGTTCAGAGTTTAGACCACTTTCTTTGTCTGA
CACTCCGGGTAACCCATGCTGGGTGCAAAATAGACTACTGAAAATTTTTTTGCTTTGTGGTTGGGACTTG
AGCCAAGGGTATAAAAGACCACCGTCCCCGAATTACCTTTCCTCTTCTTTTCTCTCTCTCCTTGTCAACT
CACACCCGAA

Supplementary file 2

T-Coffee alignment result

MSA

The multiple sequence alignment result as produced by T-coffee.

```
T-COFFEE, Version_11.00 (Version_11.00)
Cedric Notredame
SCORE=822
*
 BAD AVG GOOD
*
pTEF Yarrowia l     :  72
Yarrowia osloen     :  73
Yarrowia deform     :  74
Yarrowia galli      :  73
Yarrowia yakush     :  71
Yarrowia alimen     :  68
Yarrowia_hollan     :  70
cons                :  82

pTEF_Yarrowia_l     GGGT---------TGGCGGCG-TATTTGTGTCC-CAAAAAACAGCCCCAATTGC-C-CCAATT
Yarrowia_osloen     GGGT-C------GTGGAGGCGATATTTTCGCCCGGCGGAAACA--CACAATTGC-T-CCAATT
Yarrowia_deform     GGT----------TAGAGTCG-CTTAATCGACAATCAAAAACCAT-ACGATTGA-T-CCAATT
Yarrowia_galli      GGGG-T-------TAGAGACT-CAATATTGACAATC-AAAGTGGCCAGATTCGT-T-GCAATT
Yarrowia_yakush     GAGAGT----G-TTTGAGAGA-GAATATTGAGAATGTAAACTCGA-GCAATTGG-T-CATATT
Yarrowia_alimen     GGCC-AAAAGTCGTGCAGCCGGCAATTCCGCCA----ATTGACG--GCAATTGCGT-GCAATT
Yarrowia_hollan     AGAC-G----G-CATGTGGCC-GAAAGCA--GGGCGAAAAACAGT-GAATTCCA-TCCTAATT

cons                                 *                                *         ***


pTEF_Yarrowia_l     G-ACCCCAAATTGA----CCCAGTAGCGGGCCCAACC-CCG-GCGAGAGCCCCCTTCACCCCA
Yarrowia_osloen     G-GCGCCCAATTGA----CCCTGTAAACTCCCCAGAGCCCC-ACACCC-GCTCCCTCACCCCA
Yarrowia_deform     G-ACGTGCAATTGA----CCGGGTGGCATTTTCAGAGTCTC-ACCTTA-CCTCCCAC-CCCCA
Yarrowia_galli      G-ACCTGCAATTGA----CCCTGTAGCTTCTCCAGCTCGCC-ACCACC-CCTACATCACCCCA
Yarrowia_yakush     GCGCCCACAATTGA----CCCTACACGTCTCTCGCAGCCCC-AGAACC-ACCCAATGACCCCA
Yarrowia_alimen     G-GACGGCATTTTGCACGCCCAGCCGCCTCCCCACGTTGCCTACAACC--CCACGAGTCCCGC
Yarrowia_hollan     G-AGCACCAATTGA----CTCTGTGAGATCCTGAACCACTC-ATATCA--CACCCACACCCCA

cons                *       * **      *                                *       ***


pTEF_Yarrowia_l     CATATCAAAC--CTCCCCCGGTTCCCACACTTGCCGTTAAGGGCGTAGGGTACTGCAGTCTGG
Yarrowia_osloen     CACAACTCGT--CTCCCCCGGTTCTCACACTTGCCGTTAAGGGCGTAGGGTACTGCAGTCTGG
Yarrowia_deform     CAAAACCCCG--TTTCCCCCACCGTCACACTTGCCGTTAAGGGCGTAGGCAGCTGCAGTCTGG
Yarrowia_galli      CACAACACTG--TTCCCCCAGTTCTCACACTTGCCGTTAAGGGCGTAGGCGTCTGCAGTCTGG
Yarrowia_yakush     CACAACTCTC--TTGCCCCAGTTGTCACACTTGCCGTTAAGGGCGTAGAGTACTGCAGTCTGG
Yarrowia_alimen     CACCTCTGGGGTCGCTATTGGGGCTTGAGTTTCCCGTT-AGGGCGTAGGGTAATGCAGTCGGT
Yarrowia_hollan     CACTCAGCAC--TCTCCCCGGCACTTCAGTTTCCCGTTAAGGGCGTAGGGTACTGCAGTCTGG

cons                **                            ** ***** *********     ******* *


pTEF_Yarrowia_l     AATCTACGCTTGTTCAGACTTTGTACTAGTTTCTTTGTCTGGCCATCCGGGTAACCCATGCCG
Yarrowia_osloen     AATCTACGCTTGTTCAGACTTCGGGCCGGTTTCTTTGTCCAGCCATCCGGGTAACCCATGCCG
Yarrowia_deform     AATCTACGCTTGTTCAGAATTCGGACCTTTTTCTTTGTCCGGCCATCCGGGTAACCCATGCCG
Yarrowia_galli      AATCTACGCTTGTTCAGAATTCAGCCCTGTTTCTTTGTCCAGCCATCCGGGTAACCCATGCCG
Yarrowia_yakush     AATCTACGCATGTTCAGACTTTGGCACTGTTTCTTTGTCTGACGATCCGGGTAACCCATGCTG
Yarrowia_alimen     AAGCTACATCCATACAGAGTTTAGTCTCCTTTCTTTGTCCGCTAGGTCGGGTAACCCATGCTT
Yarrowia_hollan     AACCTACGATCGTTCAGAGTTTAGACCACTTTCTTTGTCTGACACTCCGGGTAACCCATGCTG

cons                ** ****     * **** **        **********        **************


pTEF_Yarrowia_l     GACGCAAAATAGACTACTGAAAATTTTTTTGCTT-TGTGGTTGGGACTTTAGCCAAGGGTATA
Yarrowia_osloen     GACGCAAAATAGACTACTGAAAATTTTTTTGCTT-TGTGGTTGGGACTTTAGCCAAGGGTATA
Yarrowia_deform     ACCGCAAAATAGACTACTGAAAATTTTTATGCTC-TGTGGTTGGGAGGTAGCCCCAGGGTATA
Yarrowia_galli      GACGCAAAATAGACTACTGAAAATTTTTATGCTC-TGTGGTTGGGACTTTAGCCAAGGGTATA
Yarrowia_yakush     GGCGGCAAATAGCCTTTTGAAAATTTTTTTGCCT-GGAGGTTGGGACTTTGGCCCAGGGTATA
Yarrowia_alimen     GTCGCT-TTTAGAGTCATGAAAATTTTTTTTTGCAACACCTGCGTCTTCGCTCCCAGGTATA
Yarrowia_hollan     GGTGCAAAATAGACTACTGAAAATTTTTTTGCTT-TGTGGTTGGGACTTGAGCCAAGGGTATA

cons                   *     ***  *  *********** *           ** *   *    *   ******


pTEF_Yarrowia_l     AAAGACCACCGTCCCCGAATTACCTTTCCTCTTCTTTTCTCTCTCTCCTTGT-CAACTCACAC
Yarrowia_osloen     AAAGACCACCGTCCCCGAATTACCTTTCCTCT---TTTCTCTCTCTCCTTGT-CAACTCACAC
Yarrowia_deform     AAAGACCACCGTCCCCGAATTACCTTTCCTCTTCTTTTCTCTCTCTCCTTGT-CAACTCACAC
Yarrowia_galli      AAAGACCACCGTCCCCGAATTACCTTTCCTCTTCTTTTCTCTCTCTCCTTGT-CAACTCACAC
Yarrowia_yakush     AAAGACCACCGTCCCCGAATTACCTTTCCTCTTCTTTTCTCTCTCTCCTTGTATTAGTTTCAT
Yarrowia_alimen     AAAGAGACGAGTTTCCGAATTACCTTTCTTCTTTTTTTCT---TCTCCTTGT-CAACTCACAC
Yarrowia_hollan     AAAGACCACCGTCCCCGAATTACCTTTCCTCTTCTTTTCTCTCTCTCCTTGT-CAACTCACAC

cons                *****     **  ************** ***   *****   *********   * *  **


pTEF_Yarrowia_l     CCGAA
Yarrowia_osloen     CTGAA
Yarrowia_deform     CCGAA
Yarrowia_galli      CTGAA
Yarrowia_yakush     -TCAA
Yarrowia_alimen     CTGAA
Yarrowia_hollan     CCGAA

cons                   **
```

Supplementary table 1. List of strains generated in this study

Strain number	specie	Vector used
YL_001	Yarrowia lipolytica W29	HGH pTEF-RS2
YL_005	Yarrowia lipolytica W29	NAT pTEF-RS2
CL_004	Candida oslonensis CBS 10146	-HGH-pTEF RS2
CL_006	Yarrowia deformans CBS 2071	-HGH-pTEF RS2
CL_008	Candida galli CBS 9722	-HGH-pTEF RS2
CL_010	Yarrowia yakushimensis CBS 10253	-HGH-pTEF RS2
CL_017	Candida hollandica CBS 4855	-HGH-pTEF RS2
CL_001	Candida oslonensis CBS 10146	-NAT-pTEF RS2
CL_011	Yarrowia deformans CBS 2071	NAT-pTEF RS2
CL_013	Candida galli CBS 9722	NAT-pTEF RS2
CL_015	Yarrowia yakushimensis CBS 10253	NAT-pTEF RS2
CL_003	Candida hollandica CBS 4855	-NAT-pTEF RS2
CL_019	Candida galli CBS 9722	GGE115
CL_020	Yarrowia deformans CBS 2071	GGE115
CL_021	Candida hollandica CBS 4855	GGE115
CL_022	Candida oslonensis CBS 10146	GGE115
YL_006	Yarrowia lipolytica W29	GGE115
